# SPME-GC-MS and PTR-ToF-MS Techniques for the Profiling of the Metabolomic Pattern of VOCs and GC-MS for the Determination of the Cannabinoid Content of Three Cultivars of *Cannabis sativa* L. Pollen

**DOI:** 10.3390/molecules27248739

**Published:** 2022-12-09

**Authors:** Cosimo Taiti, Elisa Masi, Vittoria Cicaloni, Vittorio Vinciguerra, Laura Salvini, Stefania Garzoli

**Affiliations:** 1 Department of Agri-Food and Environmental Science, Università di Firenze, Sesto Fiorentino, 50019 Firenze, Italy; 2 Toscana Life Sciences Foundation, 53100 Siena, Italy; 3 Department for Innovation in Biological Systems, Food and Forestry, University of Tuscia, 01100 Viterbo, Italy; 4 Department of Drug Chemistry and Technology, Sapienza University, 00185 Rome, Italy

**Keywords:** volatile compounds, cannabinoids, chromatographic and spectrometric analyses, multivariate statistical analysis

## Abstract

Considering the large number of volatile molecules that characterize *Cannabis sativa* L., adequate investigation supported by the application of robust and effective analytical methods is essential to better understand the impact of these low- and medium-molecular-weight molecules on the entire phytocomplex. This work aimed to characterize the volatile fraction of the chemical profile of three different cultivars of *Cannabis sativa* L. pollen, grown in Italy, which were thoroughly investigated by the application of two complementary techniques: SPME-GC-MS and PTR-ToF-MS. Furthermore, in order to provide more information on the chemical profile of the matrices under study, the cannabinoid content of the hexane extracts was also measured by GC-MS. Until now, no similar study, in terms of survey techniques applied, has been performed on *C. sativa* pollen. The obtained results showed a high content of volatile molecules, which differentiated the three matrices. The data relating to the content of cannabinoids were also interesting as they showed that one of the three cultivars was richer than the others. Finally, an in-depth statistical survey was performed to better compare the investigated samples and identify the molecules that most contribute to differentiating them. The findings of this study may be useful for integrating the compositional information on *C. sativa* L.

## 1. Introduction

*Cannabis sativa* L. (*C. sativa*), belonging to the Cannabaceae family, is a dioecious, annual-flowering plant, native to central temperate Asia, whose earliest use dates back about 12,000 years to China, where hemp fiber was used to make clothing, paper, sails, and ropes, while its seeds were used as food. Male and female flowers develop on separate plants. Generally, it is only at a more advanced stage of development, that is, when sexual dimorphism occurs, that it is possible to morphologically differentiate female plants from male ones; however, thanks to the use of particular molecular techniques, this differentiation is now possible even at the vegetative stage [1]. The plant height of *C. sativa* can vary from 0.2 m to 6 m, and it has palmate leaves with 3–9 linear-lanceolate leaflets. Over time, different parts of the plant have been used for medicinal and recreational purposes, religious rituals, and as a psychotropic drug. The content of secondary metabolites is very high and includes both cannabinoids and non-cannabinoids. Generally, female inflorescences are densely covered with capitate and peduncolate trichomes, where most cannabinoids and terpenoids are biosynthesized and stored. In contrast, leaves, stems, and roots, having a gradually decreasing number of trichomes, are devoid of cannabinoids. Among the cannabinoids, the key constituents of *C. sativa* are Δ-9-tetrahydrocannabinol (D9-THC), which is responsible for the psychoactive effects due to its ability to cross the blood–brain barrier because of its lipophilic structure, and cannabidiol (CBD), which is known as a non-psychoactive compound. Female cannabis inflorescences [2] have the highest level of THC, and based on this content, three types of cannabis can be distinguished: THC-dominant (chemotype I), characterized by a high THC/CBD ratio with more than 0.3% THC and less than 0.5% CBD, which are thus used to produce drugs such as marijuana and hashish; medium type (chemotype II), with a medium THC/CBD ratio (approximately equal to 1), with a high content of both CBD (more than 0.5% THC) and THC (more than 0.3% THC), and therefore with low activity; fiber type (chemotype III), called hemp, which is characterized by a low THC/CBD ratio (less than 1)—in fact, it is CBD dominant, with less than 0.3% THC, so it is not psychoactive and is therefore used for fiber and edible oil production [3,4]. Although environmental factors influence cannabinoid production during development stages in different parts of the plant [5], genetic control plays a key role [3]. Non-cannabinoid compounds belong to different chemical classes such as phenols, alkaloids, alcohols, terpenes, and others. More than 200 terpenoids have been identified in the flower and leaves of the plant, which are responsible for the fragrance of *C. sativa* [6]. Of these, limonene, myrcene, and pinene are the most common. The production of terpenoids changes significantly with environmental conditions; in fact, they are produced as a defense mechanism of the plant and their quantity increases with exposure to light (i.e., under stress conditions) [7]. The medicinal properties of cannabinoids seem to be enhanced enough to obtain excellent results in the treatment of anxious, painful, and infectious states due to the synergistic effect created between phytocannabinoids and terpenoids.

Thus, this study aimed to provide, for the first time, the identification of the volatile metabolites and cannabinoids content of three different cultivars, Skuff Mint Chocolate (SMC), Skuff CBD (SCBD), and Gelato (GEL), of *C. sativa* pollen harvested in Italy. They were obtained from greenhouse crops located in the Tuscan-Emilian Apennines (Italy), where all processing cycles are carried out strictly by hand and the entire tanning process, followed step by step, has particularly long processing times to obtain high-quality products. During the cultivation phase, a special method of controlling humidity, temperature, and carbon dioxide is used to ensure a high-quality yield. In addition, no fertilizers, pesticides, metals, and/or fertilizers are used.

In detail, SMC pollen is a cultivar obtained by beating the inflorescences with wider meshes and is characterized by a distinctive aroma that mixes tones of chocolate, mint, and pine. It has a color that turns from greenish-brown to bright green with a firm, compact texture. SCBD pollen, on the other hand, is obtained by cold beating with a 190-micron mesh and is characterized by an intense and full-bodied aroma. Finally, GEL pollen, obtained from a careful selection of home-grown cannabis light inflorescences, is characterized by a sweet and lemony scent and floral and spicy tones that make it unique in its kind.

For the purpose of this work, two complementary techniques, solid-phase microextraction–gas chromatography–mass spectrometry (SPME-GC-MS) and proton-transfer-reaction time-of-flight mass spectrometer (PTR-ToF-MS), were used; to assess the chemical profiles and identify cannabinoids, GC-detector flame ionization (GC-FID) and GC-MS were applied. Furthermore, to better compare the data obtained from chemical analyses with the aim of highlighting similarities and/or differences in chemical constituents, a thorough statistical investigation was carried out. 

To the best of our knowledge, there are no previous reports simultaneously describing the volatile profile and cannabinoid content of *C. sativa* L. pollen samples grown in Italy. 

## 2. Results

### 2.1. SPME-GC-FID Chemical Composition of Pollen

SPME sampling followed by chromatographic analyses allowed the identification of the 16 volatile components listed in Table 1. Sesquiterpenoid content exceeded monoterpenic content for all three cultivars. In particular, SCBD was characterized only by sesquiterpenes. Similarities in the chemical composition of the three investigated samples were present and concerned the most abundant components. In detail, *β*-caryophyllene was the principal component, reaching similar relative percentages (44.2% and 42.4%) in SCBD and GEL, respectively, while in SMC it exceeded 50% (53.1%). Other compounds with an almost overlapping percentage content were humulene (10.0% SCBD; 10.1% SMC; 9.3% GEL), selina 3,7-(11-diene (13.9% SCBD; 9.6% SCM; 14.7% GEL), and *β*-bisabolene (8.6% SCBD; 6.6% SMC; 6.6% GEL). On the other hand, important qualitative differences were also noted. For example, guaia-3,9-diene was present only in SCBD (22.9%) and in GEL (9.5%) and absent in SMC, while *α*-farnesene (2.4%; 2.9%) was only in SMC and GEL. Some sesquiterpenes, such as *γ*-gurjunene (6.6%), valencene (3.3%), guaia-1(10), 11-diene (1.5%), and guaiol acetate (2.1%), were detected only in GEL. Furthermore, the presence of monoterpenes characterized only SMC and GEL, even if the latter was only to a lesser extent; in fact, *β*-myrcene (5.9%), fenchone (0.5%), and *α*-terpineol (3.0%) were present in the former, while sabinene (0.8%) and *α*-terpineol (0.1%) were present in the latter with rather low relative percentages.

**Figure 1:**
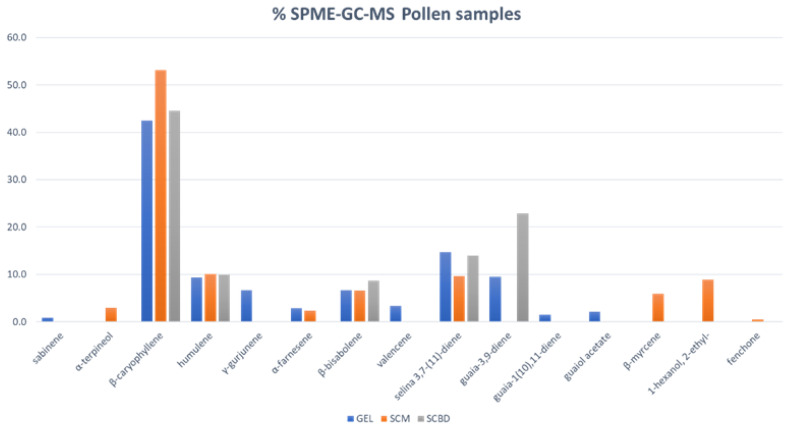
Chemical volatile percentage composition of GEL, SMC, and SCBD of *C. sativa* pollen samples.

The results reported in Table 1 are additionally displayed in a bar plot (Figure 1), where it is noticeable that *β*-caryophyllene was the most abundant component, followed by guaia-3,9-diene (present only in SCBD), selina 3,7-(11)-diene, humulene, and *β*-bisabolene.

### 2.2. GC-FID Chemical Composition of C. sativa Pollen Extracts

The chromatographic analyses performed on the hexane extracts allowed the identification of 20 components (Table 2). Among these, 19 components were identified in the GEL sample, 7 in SCBD, and only 4 in SMC. In addition to the presence of some terpene compounds, including sesquiterpenes for the most part and a few monoterpenes, the GEL extract was the sample consisting of the largest number of identified cannabinoids. In fact, in addition to cannabidiol, which reached the highest relative percentages in GEL (92.4%) and was also detected in the other two extracts (84.9% in SCM; 84.1% in SCBD), the other five cannabinoid derivatives, Δ-9-tetrahydrocannabinolo or dronabinol (2.0%), Δ-8-tetrahydrocannabidiol (0.7%), cannabichromene (0.5%), cannabinol (0.3%), and cannabigerol (0.1%), were found only in the GEL extract.

Among the sesquiterpenes, only caryophyllene oxide (0.1% in GEL; 2.8% in SMC; 2.8% in SCBD), *β*-eudesmol (0.9% in GEL; 3.6% in SMC; 2.6% in SCBD), and hexaydrofarnesyl acetone (0.2% in GEL; 2.1% in SMC; 2.2% in SCBD) were common to the three extracts, as well as the diterpene phytol (0.1% in GEL; 6.6% in SMC; 6.1% in SCBD). Other terpene compounds, including four monoterpenes (linalool, fenchol, endoborneol, and *α*-terpineol, with a percentage value ≤1.0%) and some sesquiterpenese (*β*-caryophyllene (0.7%); caryophyllene oxide (0.1%); *γ*-eudesmol (0.5%); *β*-eudesmene and nerolidol, both with a percentage content <0.1%) were detected only in GEL. Finally, *α*-bisabolol was found in GEL (1.1%) and in SCBD (1.4%), while *β*-caryophyllene (0.8%) and *γ*-eudesmol (0.6%) were present only in GEL and missing in the other extracts.

**Figure 2:**
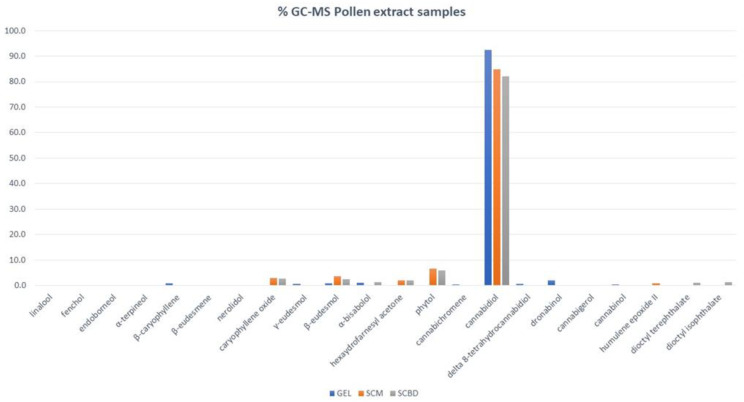
Chemical volatile percentage composition of GEL, SMC, and SCBD of *C. sativa* pollen extracts.

The results are additionally described in the bar plot in Figure 2, showing an elevated level of cannabidiol, which reached the highest relative percentages, followed byphytol, *β*-eudesmol, caryophyllene oxide, and hexaydrofarnesyl acetone.

### 2.3. PTR-ToF-MS: Determination of Volatile Compounds from C. sativa Pollen

PTR-ToF-MS analyses provided a volatile fingerprint from different *C. sativa* pollens, with high mass resolution and short acquisition times and without sample modifications. In particular, a total of 58 tentatively identified compounds in the range of measured masses (*m*/*z* = 20–300) were detected from three different cultivars of *C. sativa* pollen, and the results are reported in Table 3. Indeed, the mass spectrum observed varied for each analyzed pollen cultivar, in terms of compound typologies and concentration. As a general overview, a total of 46 different signals were detected in GEL, and 47 in SCBD, while SMC showed the higher number of signals, with a total of 57 different detected peaks. In the same way, the total emission intensity differed among the samples, with GEL showing the lowest emission intensity (~2900 ppbv), followed by SMC (~12,000 ppbv), while SCBD showed the highest (~13,500 ppbv). The emission intensity of SBCD seems to be linked to the high values of two compounds detected at *m*/*z* 33 and *m*/*z* 59, which together account for over 60% of the total issue. On the contrary, the lowest emission of GEL seems linked to the limited emission of *m*/*z* 31 and *m*/*z* 59, which were at least 10 times lower than the other two samples.

In particular, for each sample, the following compounds showed the highest emission intensity (compounds with % emission highest than 10% of total): *m*/*z* 31.018 (tentative identification as formaldehyde), *m*/*z* 33.033 (TI methanol), and *m*/*z* 59.049 (TI as acetone) in SCBD; *m*/*z* 31.018 (TI as formaldehyde) and *m*/*z* 59.049 (TI as acetone) in SMC; and *m*/*z* 31.018 (TI as formaldehyde), *m*/*z* 33.033 (TI as methanol), *m*/*z* 43.018, *m*/*z* 59.049 (TI as acetone), and *m*/*z* 61.028 (TI as acetic acid) in GEL. Each of these VOCs has a strong impact-odor, and in particular, acetic acid is linked to a strong acidic odor, which becomes a pickle-like odor in formaldehyde, while methanol and acetone are associated with a sweetish odor and with a faintly sweet pungent odor, respectively.

It is interesting to note that the SMC sample, despite showing the highest number of compounds emitted (*n* = 57), did not show the highest emission intensity. In addition, SCBD and SMC samples showed a similar total emission intensity for some volatile compounds (for example formaldehyde), and even if they seemed to have a comparable emission trend, they were characterized by very different terpene compositions. Furthermore, GEL and SMC samples showed similar emission values (%) for sesquiterpenes (*m*/*z* 205), terpenoids (*m*/*z* 153), monoterpenes (*m*/*z* 137), and many of their fragments (*m*/*z* 93, 95, 123, 135).

### 2.4. PTR-ToF-MS: Determination of Volatile Compounds from C. sativa Pollen Extracts

Headspace analysis by PTR-ToF-MS of the three *C. sativa* pollen extracts allowed the identification of 85 different peaks, which are listed in Table 4. Among them, a minimum of 61 peaks were detected in SCBD, followed by SMC with 72 different peaks, while a maximum of 83 peaks were detected in the GEL sample. In this case, the sample with a higher number of signals also showed a higher emission intensity. Thus, the total VOCs emission was higher in GEL, followed by SMC and SCBD.

Among the terpene class compounds, a great variability in the sample analysis was noticeable. Looking at all terpene compounds combined, the total emission level was similar in all samples, except for GEL, which showed the highest emission. 

### 2.5. Multivariate Metabolomics Data Analysis

**Figure 3:**
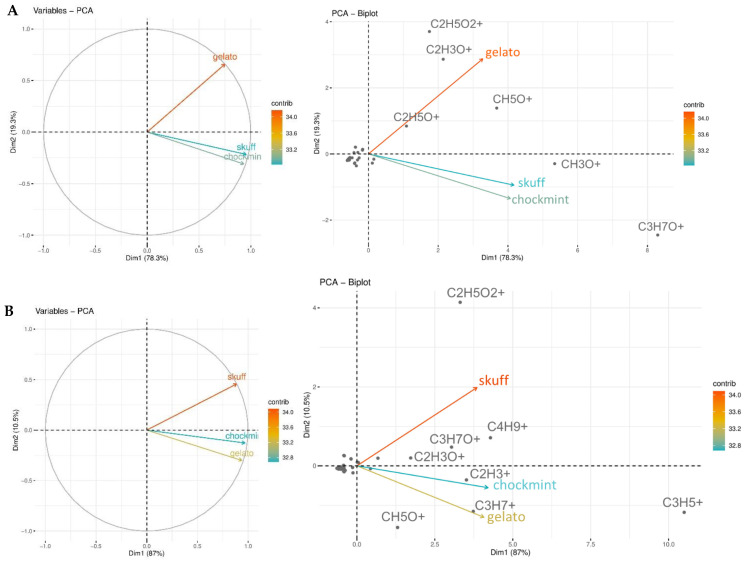
(**A**) Principal component analysis (PCA) for pollen *C. sativa* cultivars. Score plot and biplot provide information on both metabolites and samples of a data matrix to be displayed graphically. Metabolites grouped at the origin of the graph do not contribute to samples’ variability. (**B**) PCA score plot and biplot for pollen hexanoic extracts of *C. sativa* cultivars.

The obtained results from all analyses simultaneously described the volatile profile of untreated pollen samples and pollen extracts of SMC, SCBD, and GEL cultivars of *C. sativa* L. To better compare the large amount of data obtained from chemical analyses performed by PTR-ToF-MS technique and to identify similarities and/or differences in chemical constituents, a multivariate statistical investigation was carried out. In particular, an unsupervised PCA analysis was carried out to determine how metabolites differ from samples, and which compounds contribute the most to this difference. Namely, the PCA unsupervised algorithm is an orthogonal linear transformation of possibly correlated variables into a smaller number of uncorrelated variables called principal components (PCs) by revealing group structure when within-group variation is sufficiently less than between-group variation. PCA allows us to determine the contribution of the original variables to the PC model. In the case of pollen samples, the performed PCA was based on the first two principal PCs scores: PC1 explained 78.3%, and PC2 explained 19.3%. The PCA score plot (Figure 3A) showed a separation of GEL in comparison with the other cultivars. GEL was the main contributor to the samples’ variability and the most dissimilar in term of percentages of its metabolites. SCBD and SMC were clustered within the same quadrant, suggesting a similarity in the levels of their metabolites. In addition, a biplot (Figure 3A) provides information on both metabolites (displayed as points) and samples (displayed as vectors) of a data matrix. Metabolites grouped at the origin of the graph did not contribute to samples’ variability. SCBD and SMC were mostly characterized by CH_3_O^+^ (formaldehyde) and C_3_H_7_O^+^ (acetone), whereas GEL was mostly characterized by C_2_H_5_O_2_^+^ (acetic acid), C_2_H_3_O^+^ (alkyl fragment (e.g., terpenes and other compounds)), CH_5_O^+^ (methanol), and C_2_H_5_O^+^ (acetaldehyde). 

Unlike the same PTR-ToF-MS analysis applied to pollen extract samples (Figure 3B), it was found that GEL and SMC were clustered within the same quadrant, suggesting a similarity in the levels of their metabolites, while SCBD, which was the main contributor to the samples’ variability, was the most dissimilar in term of the percentage of its metabolites. PC1 explained 87.0%, and PC2 explained 10.5%. GEL and SMC were mostly characterized by C_2_H_3_^+^ (acetylene), C_3_H_7_^+^ (alkyl fragment (propene)), and C_3_H_5_^+^ (alkyl fragments). CH_5_O^+^ (methanol) was particularly represented in GEL. The main contributors to SCBD were C_2_H_5_O_2_^+^ (acetic acid), C_4_H_9_^+^ (Alkyl fragment), C_2_H_3_O^+^ (alkyl fragment (e.g., terpenes and other compounds)), and C_3_H_7_O^+^ (acetone).

**Figure 4:**
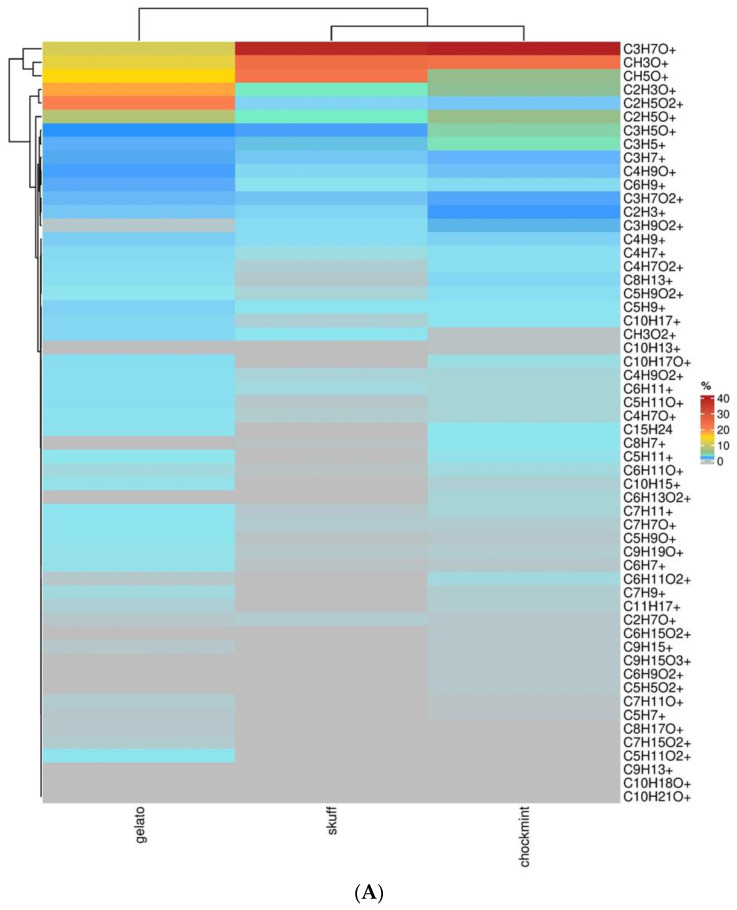

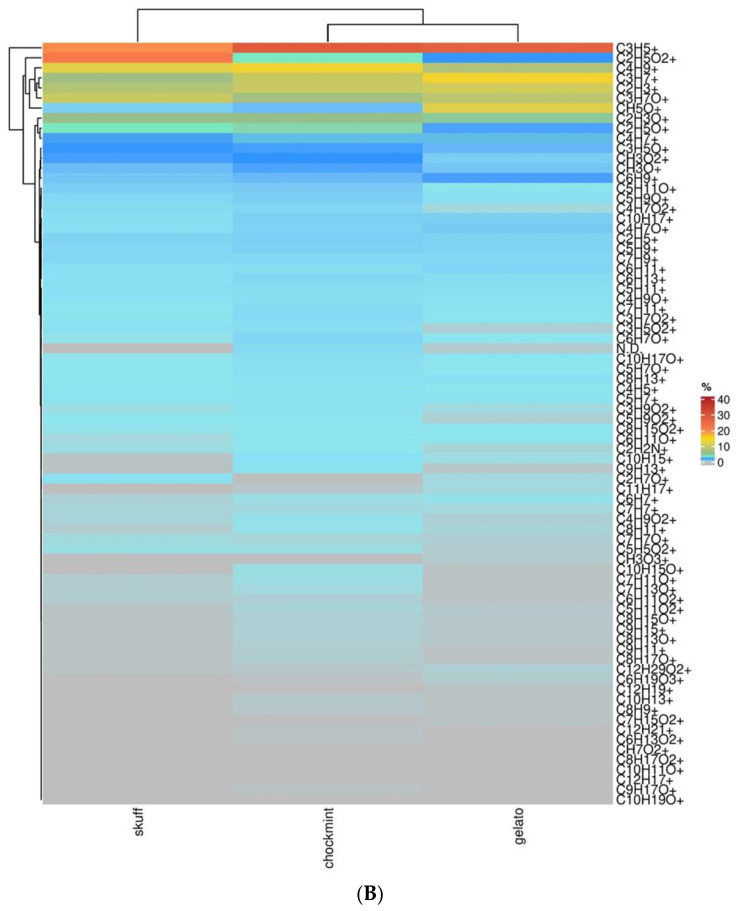
(**A**) Heatmap of metabolites percentage of the pollen samples deriving from the three cultivars. Each cultivar is indicated in the column, and every row indicates a compound. Red indicates high abundance, whereas compounds under the detection threshold are in gray. (**B**) Heatmap of metabolites percentage of the pollen hexanoic extract samples deriving from the three cultivars.

The complete list of compounds obtained by PTR-ToF-MS analysis of the pollen samples is reported in a heatmap (Figure 4A). Next to the heatmap, a hierarchical clustering (HCA) technique based on the Euclidean distance method is employed to analyze the similarities of metabolite trends in the different cultivars with a repetitive process that associates/dissociates (agglomerative/divisive methods) object by object until all are equally and completely processed [8,9]. In the first cluster, the most relevant compounds were shown to be C_3_H_7_O^+^ (acetone), CH_3_O^+^ (formaldehyde), and CH_5_O^+^ (methanol), identified in the three cultivars and sharing a common trend in which their levels were particularly elevated in SCBD. The second cluster defined by the dendrogram branch showed C_2_H_3_O^+^ (alkyl fragment (e.g., terpenes and other compounds)) and C_2_H_5_O_2_^+^ (acetic acid) as metabolites with a high level in GEL. In the third cluster, we found only the compound C_2_H_5_O^+^ (acetaldehyde), which showed a singular trend and was higher in GEL and SMC but lower in SCBD. C_3_H_5_O^+^ (alkyl fragment (e.g., hexanal/1-butanol/1-octanol)) and C_3_H_5_^+^ (alkyl fragments) were gathered in the fourth cluster, with an elevated percentage in SMC. Compounds under the detection threshold are represented in grey.

Similarly, a heatmap and HCA (Figure 4B) were performed for pollen hexanoic extract samples deriving from the same three cultivars. The first cluster included a single metabolite C_3_H_5_^+^ (alkyl fragment) that was elevated in GEL and SMC. The second cluster was represented by the six metabolites showing a significant composition percentage (C_2_H_5_O_2_^+^ (acetic acid), C_4_H_9_^+^ (alkyl fragment), C_3_H_7_+ (alkyl fragment (propene)), C_2_H_3_^+^ (acetylene), C_3_H_7_O^+^ (acetone) and CH_5_O^+^ (methanol)). In the third cluster, the following metabolites were noticeable: C_2_H_3_O^+^ (alkyl fragment (e.g., terpenes and other compounds)), C_2_H_5_O^+^ (acetaldehyde), and C_4_H_7_^+^ (C4 aldehydes fragment).

**Figure 5:**
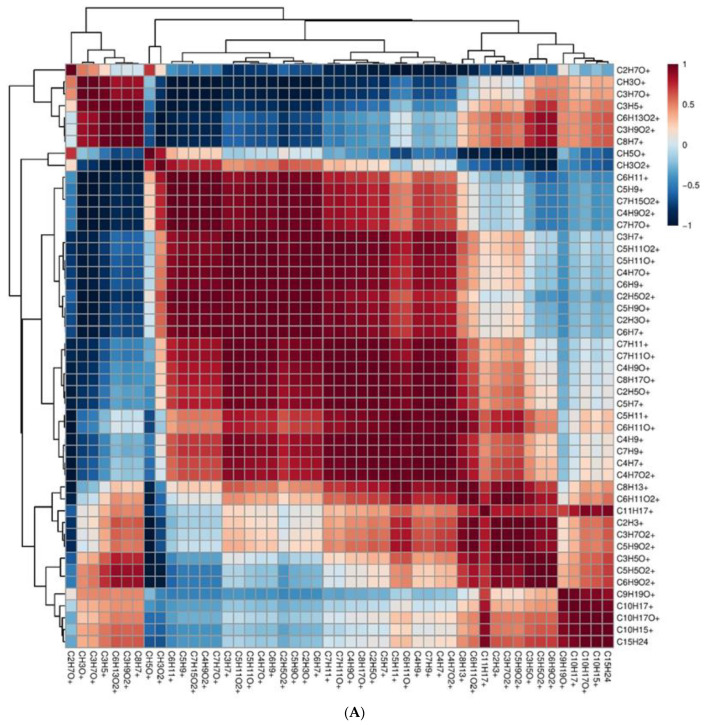

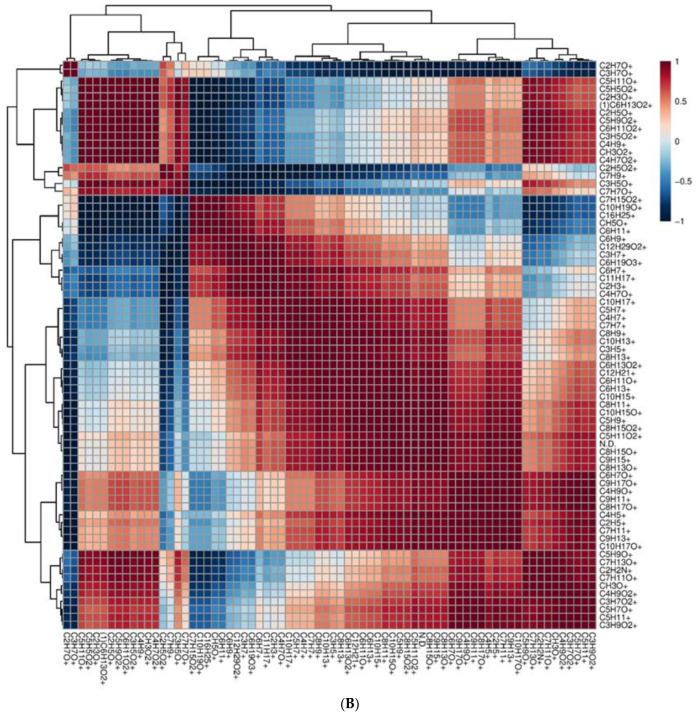
(**A**) Correlation matrix based on Pearson correlation coefficient among different compounds deriving from pollen with dendrogram. (**B**) Correlation matrix based on Pearson correlation coefficient among different compounds deriving from pollen extracts with dendrogram.

Furthermore, Pearson’s correlation analysis was performed among various metabolites of *C. sativa* pollen. In Figure 5A, the complete dataset of pollen compounds is explored with a correlation matrix, a table representing correlation coefficients between variables. The dendrogram next to the correlation matrix clustered compounds sharing a similar correlation trend. The most important direct correlations were between the following sub-groups:

1° group: CH_3_O^+^ (formaldehyde), C_3_H_7_O^+^ (acetone), C_3_H_5_^+^ (fragments), C_6_H_13_O_2_^+^ (hexanoic acid/methyl valerate), C_3_H_9_O_2_+ (propylene glycol), C_8_H_7_^+^ (fragments); and the 2° group: C_8_H_13_^+^ (sesquiterpenes/terpenes fragments), C_6_H_11_O_2_^+^ (γ-hexalactone), C_11_H_17_^+^ (sesquiterpene fragments), C_2_H_3_^+^ (acetylene), C_3_H_7_O_2_^+^ (methyl acetate/propanoates), C_5_H_9_O_2_^+^ (hexanal), C_3_H_5_O^+^ (alkyl fragment (e.g., hexanal/1-butanol/1-octanol)), C_5_H_5_O_2_^+^ (furfural), C_6_H_9_O_2_^+^ (dimethyl-furanone/methyl-cyclopentanedione/cyclotene), C_9_H_19_O^+^ (nonanal), C_10_H_17_^+^ (monoterpenes), C_10_H_17_O^+^ (terpenoids), C_10_H_15_^+^ (*p*-cymene), and C_15_H_25_ (sesquiterpenes).

Another significant correlation emerged between the 1°subset C_3_H_7_^+^ (alkyl fragment (propene)), C_5_H_11_O_2_^+^ (butyl formate), C_5_H_11_O^+^ (pentanal), C_4_H_7_O^+^ (2-butenal), and C_6_H_9_^+^ (C6 and terpenes fragments) and the 2° subgroup C_7_H_11_^+^ (terpenes fragments), C_7_H_11_O^+^ (dimethyl-2-cyclopenten-1-one), C_4_H_9_O^+^ (2-butanone/isobutyraldehyde), C_8_H_17_O^+^ (methyl hexyl ketone), C_2_H_5_O^+^ (acetaldehyde), C_5_H_7_^+^ (terpenes fragments), C_5_H_11_^+^ (3-methyl-1-butanol/alcohol fragment), C_6_H_11_O^+^ (hexenal/methyl-pentenone), C_4_H_9_^+^ (alkyl fragment), C_7_H_9_^+^ (terpenes fragments), C_4_H_7_^+^ (C4 aldehydes fragment), and C_4_H_7_O_2_^+^ (2,3-butandione/diacetyl). 

Similarly, a correlation matrix was also performed for pollen hexanoic extract samples (Figure 5B). The most important direct correlations resulted from the following sub-groups:

1° group: C_5_H_11_O^+^ (pentanal), C_5_H_5_O_2_^+^ (pentanal), C_2_H_3_O^+^ (alkyl fragment (e.g., terpenes and other compounds)), C_6_H_13_O_2_^+^ (hexanoic acid/methyl valerate), C_2_H_5_O^+^ (acetaldehyde), C_5_H_9_O_2_^+^ (hexanal), C_6_H_11_O_2_^+^ (γ-hexalactone), C_3_H_5_O_2_^+^, C_4_H_9_^+^ (alkyl fragment), CH_3_O_2_^+^ (formic acid/formates), and C_4_H_7_O_2_^+^ (2,3-butandione/diacetyl); and 2° group: C_5_H_9_O^+^ (methyl-butenal/pentenone), C_7_H_13_O^+^ (identification not assigned), C_7_H_11_O^+^ (dimethyl-2-cyclopenten-1-one), CH_3_O^+^ (formaldehyde), C_4_H_9_O_2_^+^ (ethyl acetate/methyl-propanoate), C_3_H_7_O_2_^+^ (methyl acetate/propanoates), C_5_H_7_O^+^ (3-methyl furan), C_5_H_11_^+^ (3-methyl-1-butanol/alcohol fragment), and C_3_H_9_O_2_^+^ (propylene glycol).

The other significant direct correlation was between 1° subgroup C_8_H_11_^+^ (terpenes fragments), C_10_H_15_O^+^ (monoterpenes oxygenate), C_5_H_9_^+^ (isoprene/terpenes fragments), C_8_H_15_O_2_^+^ (lactone compound), C_5_H_11_O_2_^+^ (butyl formate), C_8_H_15_O^+^ (1-Octen-3-one), C9H15^+^ (sesquiterpene fragments), and C_8_H_13_O^+^ ((E)-2-octenal) and 2° group C_6_H_7_O^+^ (phenol), C_4_H_9_O^+^ (2-butanone/isobutyraldehyde), C_9_H_11_^+^ (terpenes fragments), C_8_H_17_O^+^ (methyl hexyl ketone), C_4_H_5_^+^ (C4 fragments), C_2_H_5_^+^ (fragments), C_7_H_11_^+^ (terpenes fragments), C_9_H_13_^+^ (sesquiterpene fragments), and C_10_H_17_O^+^ (terpenoids).

## 3. Discussion

In this work, for the first time, the volatolomic profile of pollen from three different cultivars of *C. sativa* L. was investigated by the application of two complementary techniques, SPME-GC-MS and ToF-PTR-MS, to highlight VOCs that are specific to the different genotypes. In general, both techniques require small amounts of sample to be analyzed, without the need for any treatment and with a short extraction time. With PTR-ToF-MS, it is possible to obtain in real time the whole volatile spectrum (including low-molecular-weight compounds) with high sensitivity and high time resolution, although some compounds are ambiguously identified [10,11,12]. On the other hand, the SPME technique, although its limitation is linked to the displacement effect of analytes with a lower affinity with the type of used fiber, is ideal for MS applications as it combines simple and efficient sample preparation with versatile and sensitive detection [13,14]. Therefore, using a combined approach, the present study represents a first step towards a better comprehension of the molecular composition of cannabis pollen. [15].

The obtained results highlighted the presence of a consistent number of volatile molecules whose content varied both qualitatively and quantitatively between the analyzed cultivars.

Except for a recent work aimed at a comparative evaluation on the emission of volatiles from pollen and entire male and female plants of two *C. sativa* variants, Northern Lights and Hawaian Indica [16], only two other older works report a composition study conducted on pollen. In the first, flavonoids and cannabinoids were detected in the pollen obtained from a Mexican variety of *C. sativa* [17], and in the other, the content of cannabinoids and nitrogenous substances of the pollen of *C. sativa* cultivated under artificial climatic conditions in France [18] was determined. 

The number, as well as the type, of identified volatiles in the pollen can vary greatly depending on the used techniques for the detection of the same, as well as on the different plant preparations [19]. In fact, contrary to our results obtained by SPME-GC-MS, where sesquiterpenes were the main constituents, Rothschild et al. [16], who had used the GC-MS technique preceded by a process of capturing the volatiles using cartridges, reported two monoterpenes, *β*-myrcene and terpinolene, and (E)-*β*-ocimene as the most abundant compounds.

In our investigation, for the first time, thanks to the sophisticated analytical techniques applied, we were able to characterize and highlight the qualitative and quantitative differences between the three *C. sativa* pollen cultivars. In general, considering that the aroma is characterized by a mixture of many volatile compounds, specific to species and sometimes for plant varieties [20], the great variability observed among our investigated samples is not surprising. The terpene compounds (terpenoids, monoterpenes, sesquiterpenes, di- and triterpenes) produced in hemp through secondary metabolism provide unique aromas specific for each cannabis variety [21], thus representing a very important chemical group that is useful to differentiate samples from each other, both for types and emission intensity. Indeed, many of the terpenes identified in the headspace were not found in all samples or they had a very different emission rate. For example, SMC pollen showed a more complex and the highest emission of terpenes compared to the other two samples, both for the number of compounds and for their total emission. In fact, the total terpenes emission (which includes monoterpenes, oxygenated terpenes, and sesquiterpenes) was ~209 ppbv in SMC, ~112 ppbv in GEL, and significantly lower in SCBD (~61 ppbv). Furthermore, following the pattern fragmentation of terpenes compounds proposed and applied in previous works [22,23,24,25,26], it was possible to note how GEL and SMC had a more similar terpene profile than SCBD. Therefore, as reported elsewhere [16], the compositional difference of *C. sativa* pollens is mainly linked to terpenes compounds that are produced between the flowering and maturity of the plants.

From the obtained data, and thanks to the analyses carried out on the extracts, it emerged that among the terpenes class compounds, there was a great variability in the samples. Moreover, to compare the results of the untreated pollen analysis with that of the extract, it was possible to observe, also by means of a multivariate statistical analysis, how it had changed drastically. 

Regarding the cannabinoid content determined on the hexane extracts of the three pollen cultivars, contrary to a previous work, where Δ9-tetrahydrocannabinol was the most abundant compound found in the hexane extracts of pollen grains from *C. sativa* L. cultivated and collected in Mississippi [17], our analyses showed a high cannabidiol content, with a relative percentage value comparable between the extracts. The other cannabinoids were instead detected only in the GEL extract sample.

## 4. Materials and Methods

### 4.1. Materials

All *C. sativa* pollen blocks (5.0 g each) collected from plants of the three cultivars were a kind gift of Appennino Farm—Gaggio Montano (BO) 40041, Italy.

For extractions, HPLC-grade hexane were purchased from Sigma-Aldrich (Steinheim, Germany).

### 4.2. Extraction Process

First, 2.0 g of each pollen variety was weighed in a flask and extracted with 5.0 mL × 3 of hexane overnight at room temperature. The three extracts were filtered, combined, and dried under reduced pressure at 30 °C to obtain, respectively, 143.0 mg (GEL, 7.15%), 7.0 mg (SCBD, 0.35%), and 9.0 mg (SMC, 0.45%). The residual pollen was subsequently extracted with 5.0 mL × 2 of methanol to afford, respectively, 207.0 mg (GEL, 10.35%), 290.0 mg (SCBD, 14.50%), and 307.0 mg (SMC, 19.50%).

### 4.3. SPME Sampling

The volatile chemical composition of the *C. sativa* pollen was performed by SPME sampling technique following Cicaloni et al. [27], with some modifications. About 2 g of each variety were placed inside a 7 mL glass vial with PTFE-coated silicone septum. The extraction process of volatiles was carried out on an SPME device from Supelco (Bellefonte, PA) with 1 cm fiber coated with 50/30 μm DVB/CAR/PDMS (divinylbenzene/carboxen/polydimethylsiloxane). Before use, the fiber was conditioned at 270 °C for 30 min. The equilibration time for all samples of pollen was obtained by heating to 60 °C for 10 min. After this time, the fiber was exposed to the headspace of the samples for 30 min at 60 °C to capture and concentrate the volatiles. Lastly, the analytes were desorbed thermally in a GC injector maintained at 250 °C for 2 min in split mode.

### 4.4. GC-MS Analysis of C. sativa Pollen

The analyses of pollen samples were carried out on a Clarus 500 model Perkin Elmer (Waltham, MA, USA) gas chromatograph coupled with a mass spectrometer equipped with an FID (flame detector ionization). The capillary column was a Varian Factor Four VF-1. To characterize the volatile composition of pollen samples, the oven conditions were set as follows: from 50 °C to 220 °C at 6°/min, and finally held for 15 min. Helium was used as carrier gas at a constant rate of 1 mL/min. MS scans were recorded within the range 35–500 *m*/*z* using EI ionization (energy 70 eV). Identification of compounds was based on the comparison of the mass spectra of pure components stored in the Wiley 2.2 and Nist 02 libraries database and on the comparison of the linear retention indices (LRIs) calculated using a series of alkane standards (C_8_–C_25_ n-alkanes) with the available retention data reported in the literature. The relative proportions of the constituents were expressed as percentages and were obtained by FID peak-area normalization (mean of three replicates) without the use of an internal standard or any factor correction.

### 4.5. GC-MS Analysis of C. sativa Pollen Extracts

To determine the cannabinoid content of pollen samples, each hexanoic extract (1 µL) was injected manually at 270 °C into the GC injector, and the injector split ratio was 1:20. The used column was the same, while the GC oven was set differently: initially at 170 °C, then increased to 210 °C at 4/min and held for 2 min, then increased to 250 °C at 4 °C/min and held for 15 min. The mass spectrometer operated at the same conditions used for the untreated pollen, and the identification and quantification of the detected compounds were performed as described in the previous Section 4.4.

### 4.6. PTR-ToF-MS Analysis of Pollen and Extracts

All the measurements were carried out within a climatized room (25 °C, 90% RH) by direct injection using a commercial PTR-ToF-MS—model 8000—(Ionicon Analytik GmbH, Innsbruck, Austria), with its settings in the standard configuration. This tool is able to detect molecules with detection limits lower than parts-per-trillion by volume (pptV) range [28], thereby providing quantitative and qualitative information of VOCs. Three different samples of powder pollen were analyzed in triplicate (4 g of pollen for each replicate), and then the pollen extracts were also analyzed. For the pollen analyses, each sample was put into a ¾ L glass jar provided with a cover with two holes, connected (with Teflon tubes) to a zero-air generator (PeakScientific, Glasgow, Scotland) and to the PTR instrument, respectively. Each pollen powder sample was incubated exactly and analyzed for 120 s. For the pollen extract analyses, 2 mL of sample was placed inside a 250 mL Pyrex glass jar, which was hermetically sealed. Subsequently, the sample was placed on a round electric heating plate set at 110 °C and equilibrated for 15 min, in order to allow VOCs from the liquid cannabis extract to enter the headspace. Once the pollen extract sample was equilibrated, it was analyzed for 300 s. Due to the limited extract content, no replicate was performed.

Before analyzing each sample, the jar was flushed with cleaned air (provided by zero-air generator) in order to remove all the VOCs accumulated in the head space during the time between sample preparation and analysis. Thereafter, each sample measurement was done using a flow rate of 0.50 L per minute (lpm), and the expelled headspace was continuously replaced with clean air. 

The tool was set with the following conditions: 60 °C drift and inlet tube temperature, 2.2 mbar drift pressure, and 594 V drift voltage, leading to an E/N ratio of about 124 Townsend (Td; 1 Td = 10–17 V cm^−2^), where E corresponds to the electric field strength and N corresponds to the gas number density. Mass spectra were recorded in the mass-to-charge (*m*/*z*) range between 20 and 220, with one mass spectrum acquired every second (the spectra image is reported in Supplementary Materials). Once the runs were recorded, the internal calibration was performed off-line following the procedure described by Fabris et al. [29] with a three-point calibration (*m*/*z* 29.997, *m*/*z* 59.049, *m*/*z* 180.937). Raw data were recorded by TOFDAQ v.183 data acquisition software (Tofwerk AG, Thun, Switzerland). The emission intensity of each *m*/*z* detected was expressed as concentration in cps and was then converted to ppbv (parts per billion by volume). Next, a noise reduction of the dataset was done by eliminating peaks whose average concentrations were under 0.5 ppb. Finally, peak identification was carried out on the basis of the high-accuracy signal provided by the ToF and by combining data from GC-MS Cannabis literature together with the PTR literature [23,24,25,26].

### 4.7. Statistical Analysis

To perform a statistical analysis of the obtained results from PTR-ToF-MS analyses, the data matrix was imported into the MetaboAnalyst 5.0 online platform [30] and graphically displayed by using several R packages (“ComplexHeatmap” version 2.11.1, “Circlize’ version 0.4.13, “ColorRamps” version 2.3, and “FactoMineR” version 2.6). To provide an exploratory data analysis for both pollens and hexanoic extracts, principal component analysis (PCA) was applied to cluster features into subgroups based on commonality and to report the weight of each component to the clusterization. It is a preliminary step in a multivariate analysis to provide an unsupervised overview of the samples. An unsupervised PCA analysis was carried out to determine how metabolites differ from each other, and which compounds contribute the most to this difference. Additionally, a hierarchical cluster analysis (HCA) was performed to obtain a dendrogram of pollen and hexanoic extracts, based on Euclidean distance, using the metabolome datasets. To visualize the variations in potential markers, heatmaps were plotted for separating samples and metabolites into different groups. To measure the linear correlation between metabolites in the datasets, a correlation matrix based on Pearson correlation coefficient was performed. The result has a value between −1 (strong inverse correlation) and 1 (strong direct correlation).

## 5. Conclusions

In the present study, a deep characterization of the volatile chemical composition of pollen from three different hemp varieties by means SPME-GC-MS analysis was carried out. A large number of compounds belonging to different chemical classes were identified. Overall, the results obtained and interpreted thanks to statistical analysis showed that there are differences in composition between the cultivars; in fact, from the PTR-ToF-MS data derived from untreated pollen, GEL and SMC had a more similar terpene profile than SCBD. This trend was reversed when the analyses were conducted on the extracts. From the SPME-GC-MS data, it was evident that GEL, from a qualitative point of view, was the richest sample in sesquiterpene compounds and, also, the richest in cannabinoids. In conclusion, the investigative analysis conducted on *C. sativa* L. pollens is undoubtedly important in the field of chemistry of natural products, and the results obtained, given the nature of the numerous secondary metabolites, are of considerable relevance for a better understanding of the phytochemical composition of *C. sativa* L.

## Figures and Tables

**Table 1:** Chemical volatile composition (percentage mean value ± standard deviation) of *C. sativa* pollen.

N°	Component ^1^	LRI ^2^	LRI ^3^	SCBD ^4^	SMC ^5^	GEL ^6^
1	sabinene	968	970	-	-	0.8 ± 0.02
2	*β*-myrcene	990	987	-	5.9 ± 0.06	-
3	1-hexanol, 2-ethyl-	1041	1037	-	8.8 ± 0.07	-
4	fenchone	1082	1080	-	0.5 ± 0.02	-
5	*α*-terpineol	1186	1183	-	3.0 ± 0.06	0.1 ± 0.02
6	*β*-caryophyllene	1445	1440	44.2 ± 0.02	53.1 ± 0.10	42.4 ± 0.07
7	humulene	1476	1473	10.0 ± 0.04	10.1 ± 0.02	9.3 ± 0.04
8	*γ*-gurjunene	1474	1479	-	-	6.6 ± 0.02
9	*α*-farnesene	1490	1496	-	2.4 ± 0.02	2.9 ± 0.06
10	*β*-bisabolene	1505	1501	8.6 ± 0.04	6.6 ± 0.02	6.6 ± 0.03
11	valencene	1517	1515	-	-	3.3 ± 0.02
12	selina 3,7-(11)-diene	1543	1540	13.9 ± 0.03	9.6 ± 0.02	14.7 ± 0.02
13	guaia-3,9-diene	1560	1556	22.9 ± 0.10	-	9.5 ± 0.03
14	guaia-1(10), 11-diene	1580	*	-	-	1.5 ± 0.02
15	guaiol acetate	1715	1712	-	-	2.1 ± 0.06
	SUM			99.6	100.0	99.8
	monoterpenes			-	9.4	0.9
	sesquiterpenes			99.6	81.8	98.9
	others			-	8.8	

^1^ The components are reported according to their elution order on apolar column; ^2^ linear retention indices measured on apolar column; ^3^ linear retention indices from literature; ^4^ SCBD: Skuff CBD pollen hemp. components; ^5^ SMC: Skuff Chocolate Mint pollen hemp components; ^6^ GEL: Gelato pollen hemp components; * LRI not available; -: not detected; detection limit = 0.5 ppb.

**Table 2:** Chemical composition (percentage mean value ± standard deviation) of *Cannabis sativa* L. pollen extracts.

N°	Component ^1^	LRI ^2^	LRI ^3^	SCBD ^4^	SMC ^5^	GEL ^6^
1	linalool	1090	1095	-	-	0.1 ± 0.02
2	fenchol	1105	1100	-	-	tr
3	endoborneol	1165	1160	-	-	tr
4	*α*-terpineol	1186	1183	-	-	0.1 ± 0.04
5	*β*-caryophyllene	1445	1440	-		0.7 ± 0.05
6	*β*-eudesmene	1487	1483	-	-	tr
7	nerolidol	1570	1565	-	-	tr
8	caryophyllene oxide	1579	1580	2.8 ± 0.04	2.8 ± 0.02	0.1 ± 0.02
9	humulene epoxide II	1610	*	0.8 ± 0.02	-	-
10	*γ*-eudesmol	1625	1630	-	-	0.5 ± 0.02
11	*β*-eudesmol	1655	1652	2.6 ± 0.05	3.6 ± 0.03	0.9 ± 0.02
12	*α*-bisabolol	1671	1665	1.4 ± 0.04	-	1.1 ± 0.02
13	hexaydrofarnesyl acetone	1852	1846	2.2 ± 0.02	2.1 ± 0.02	0.2 ± 0.02
14	phytol	2105	2101	6.1 ± 0.06	6.6 ± 0.02	0.1 ± 0.02
15	cannabichromene	2350	*	-	-	0.5 ± 0.02
16	cannabidiol	2510	*	84.1 ± 0.04	84.9 ± 0.07	92.4 ± 0.12
17	Δ-8-tetrahydrocannabidiol	2530	2525	-	-	0.7 ± 0.03
18	Δ-9-tetrahydrocannabinolo	2550	*	-	-	2.0 ± 0.02
19	cannabinol	2562	*	-	-	0.3 ± 0.03
20	cannabigerol	2577	*	-	-	0.1 ± 0.02
	SUM			100.0	100.0	99.8
	terpenoids			-	-	0.2
	sesquiterpenoids			9.0	8.5	3.5
	diterpenoids			6.1	6.6	0.1
	cannabinoids			84.1	84.9	96.0
	others			0.8	-	-

^1^ The components are reported according to their elution order on apolar column; ^2^ linear retention Indices measured on apolar column; ^3^ linear retention indices from literature; ^4^ SCBD: Skuff CBD pollen extract components; ^5^ SMC: Skuff Chocolate Mint pollen extract components; ^6^ GEL: Gelato pollen extract components; * LRI not available; tr: (mean value < 0.1%); -: not detected; detection limit = 0.5 ppb.

**Table 3:** Chemical composition (percentage emission for each *m*/*z* detected) of *C. sativa* pollen identified via PTR-ToF-MS. Data are expressed as % on the total.

*m*/*z*	Formula	Tentative Identification ^1^	SCBD (%)	SMC (%)	GEL (%)
27.022	C_2_H_3_^+^	acetylene	0.46 ± 0.02	1.75 ± 0.05	0.76 ± 0.03
31.018	CH_3_O^+^	formaldehyde	23.92 ± 5.50	23.32 ± 4.23	12.58 ± 4.75
33.033	CH_5_O^+^	methanol	22.37 ± 0.79	5.25 ± 0.58	14.81 ± 1.27
41.038	C_3_H_5_^+^	alkyl fragments	2.54 ± 0.60	3.45 ± 0.54	1.30 ± 0.26
43.018	C_2_H_3_O^+^	alkyl fragment (e.g., terpenes and other compounds)	3.11 ± 0.35	4.86 ± 0.29	17.93 ± 0.65
43.054	C_3_H_7_^+^	alkyl fragment (e.g., propene)	0.78 ± 0.04	1.20 ± 0.25	2.30 ± 0.02
45.033	C_2_H_5_O^+^	acetaldehyde	2.99 ± 0.23	5.63 ± 1.89	7.84 ± 0.46
47.012	CH_3_O_2_^+^	formic acid/formates	0.14 ± 0.02	0.02 ± 0.01	0.39 ± 0.07
47.049	C_2_H_7_O^+^	ethanol	0.04 ± 0.01	0.03 ± 0.01	0.03 ± 0.01
55.054	C_4_H_7_^+^	C4 aldehydes fragment	0.08 ± 0.01	0.26 ± 0.05	0.36 ± 0.08
57.033	C_3_H_5_O^+^	alkyl fragment (e.g., hexanal/1-butanol/1-octanol)	1.61 ± 0.25	4.11 ± 0.32	1.92 ± 0.59
57.069	C_4_H_9_^+^	alkyl fragment	0.27 ± 0.07	0.50 ± 0.31	0.63 ± 0.18
59.049	C_3_H_7_O^+^	acetone	38.70 ± 2.25	41.19 ± 7.19	10.80 ± 1.92
61.028	C_2_H_5_O_2_^+^	acetic acid	0.45 ± 0.05	0.76 ± 0.23	20.25 ± 5.11
67.054	C_5_H_7_^+^	terpenes fragments	0.01 ± 0.00	0.02 ± 0.01	0.03 ± 0.01
69.069	C_5_H_9_^+^	isoprene/terpenes fragments	0.12 ± 0.01	0.12 ± 0.02	0.52 ± 0.04
71.049	C_4_H_7_O^+^	2-butenal	0.04 ± 0.01	0.06 ± 0.01	0.15 ± 0.04
71.086	C_5_H_11_^+^	3-methyl-1-butanol/alcohol fragment	0.01 ± 0.00	0.09 ± 0.01	0.10 ± 0.04
73.064	C_4_H_9_O^+^	2-butanone/isobutyraldehyde	0.40 ± 0.01	0.92 ± 0.05	1.63 ± 0.2
75.044	C_3_H_7_O_2_^+^	methyl acetate/propanoates	0.86 ± 0.06	1.47 ± 0.21	1.08 ± 0.16
77.059	C_3_H_9_O_2_^+^	propylene glycol	0.29 ± 0.10	2.40 ± 0.70	0.03 ± 0.01
79.054	C_6_H_7_^+^	C6 fragments	0.02 ± 0.01	0.03 ± 0.01	0.09 ± 0.02
81.069	C_6_H_9_^+^	C6 and terpenes fragments	0.17 ± 0.05	0.33 ± 0.07	1.35 ± 0.23
83.086	C_6_H_11_^+^	C6 compounds/hexenol fragment	0.07 ± 0.02	0.06 ± 0.01	0.21 ± 0.01
85.065	C_5_H_9_O^+^	methyl-butenal/pentenone	0.02 ± 0.01	0.11 ± 0.02	0.11 ± 0.01
87.044	C_4_H_7_O_2_^+^	2,3-butandione/diacetyl	0.05 ± 0.01	0.30 ± 0.01	0.30 ± 0.05
87.080	C_5_H_11_O^+^	pentanal	0.03 ± 0.01	0.23 ± 0.01	0.23 ± 0.01
89.059	C_4_H_9_O_2_^+^	ethyl acetate/methyl-propanoate	0.06 ± 0.03	0.23 ± 0.02	0.23 ± 0.05
93.069	C_7_H_9_^+^	terpenes fragments	0.01 ± 0.00	0.07 ± 0.01	0.07 ± 0.01
95.086	C_7_H_11_^+^	terpenes fragments	0.03 ± 0.01	0.11 ± 0.01	0.11 ± 0.01
97.069	C_5_H_5_O_2_^+^	furfural	0.00	0.03 ± 0.01	0.00
99.080	C_6_H_11_O^+^	hexenal/methyl-pentenone	0.02 ± 0.00	0.07 ± 0.01	0.07 ± 0.00
101.060	C_5_H_9_O_2_^+^	hexanal	0.06 ± 0.01	0.22 ± 0.03	0.11 ± 0.02
103.054	C_8_H_7_^+^	fragments	0.02 ± 0.01	0.10 ± 0.02	0.00
103.075	C_5_H_11_O_2_^+^	butyl formate	0.00	0.01 ± 0.00	0.11 ± 0.00
107.049	C_7_H_7_O^+^	benzaldehyde	0.04 ± 0.01	0.04 ± 0.01	0.12 ± 0.00
109.101	C_8_H_13_^+^	sesqui/terpenes fragments	0.03 ± 0.01	0.40 ± 0.07	0.20 ± 0.01
111.080	C_7_H_11_O^+^	dimethyl-2-cyclopenten-1-one	0.01 ± 0.00	0.02 ± 0.00	0.04 ± 0.00
113.059	C_6_H_9_O_2_^+^	n.a.	0.01 ± 0.00	0.03 ± 0.01	0.01 ± 0.01
115.054	C_6_H_11_O_2_^+^	C9-aromatics	0.01 ± 0.00	0.07 ± 0.03	0.03 ± 0.01
117.091	C_6_H_13_O_2_^+^	hexanoic acid/methyl valerate	0.01 ± 0.00	0.03 ± 0.00	0.00
119.085	C_9_H_11_^+^	terpenes fragments	0.00	0.02 ± 0.01	0.00
121.101	C_9_H_13_^+^	sesquiterpene fragments	0.00	0.01 ± 0.00	0.00
123.116	C_9_H_15_^+^	sesquiterpene fragments	0.00	0.03 ± 0.01	0.03 ± 0.00
129.127	C_8_H_17_O^+^	methyl hexyl ketone	0.00	0.01 ± 0.00	0.03 ± 0.00
131.001	n.a.	n.a.	0.02 ± 0.01	0.05 ± 0.002	0.00
131.106	C_7_H_15_O_2_^+^	eptanoic acid	0.00	0.00	0.04 ± 0.02
133.101	C_10_H_13_^+^	*p*-cymenene/fragm	0.00	0.02 ± 0.00	0.00
135.116	C_10_H_15_^+^	*p*-cymene	0.01 ± 0.00	0.05 ± 0.02	0.09 ± 0.01
137.132	C_10_H_17_^+^	monoterpenes	0.05 ± 0.02	0.12 ± 0.04	0.40 ± 0.05
139.113	C_9_H_14_O^+^	nopinone/isophorone	0.00	0.03 ± 0.00	0.00
143.143	C_9_H_19_O^+^	nonanal	0.03 ± 0.01	0.05 ± 0.01	0.09 ± 0.03
149.012	n.a.	n.a.	0.01 ± 0.00	0.04 ± 0.01	0.00
149.140	C_11_H_17_^+^	sesquiterpene fragments	0.00	0.04 ± 0.01	0.05 ± 0.01
153.127	C_10_H_17_O^+^	terpenoids	0.01 ± 0.00	0.09 ± 0.02	0.27 ± 0.11
155.143	C_10_H_19_O^+^	terpenoids (e.g., terpineol/fenchyl alcohol)	0.00	0.01 ± 0.00	0.00
157.158	C_10_H_21_O^+^	decanal/terpenoids (e.g., menthol)	0.00	0.01 ± 0.00	0.00
205.195	C_15_H_25_	sesquiterpenes	0.01 ± 0.00	0.14 ± 0.03	0.16 ± 0.05
		number of signals detected	47	56	46
		total VOCs emission (ppbv)	13586.5 ± 705.3	11918.12 ± 1304.4	2970.1 ± 183.5
		terpenes total emission (ppbv)	61.12 ± 5.9	209.41 ± 19.3	111.98 ± 7.9

*m*/*z*: mass/charge ratios; formula: chemical formula for each VOCs species; ^1^ tentative identification of each signals detected: the terpene identification was based on pattern fragmentations proposed for the PTR instrument; 0.00: not detected; n.a.: identification not assigned; detection limit = 0.5 ppb.

**Table 4:** Chemical composition (percentage emission for each *m*/*z* detected) of *Cannabis sativa* pollen extracts identified via PTR-ToF-MS. Data are expressed as % on the total.

*m*/*z*	Formula	Tentative Identification ^1^	SCBD (%)	SMC (%)	GEL (%)
27.022	C_2_H_3_^+^	acetylene	7.78	9.54	10.37
29.038	C_2_H_5_^+^	fragments	0.48	0.56	0.49
31.018	CH_3_O^+^	formaldehyde	1.00	1.56	0.77
33.033	CH_5_O^+^	methanol	0.59	1.00	11.55
41.038	C_3_H_5_^+^	alkyl fragments	19.33	28.02	26.69
43.018	C_2_H_3_O^+^	alkyl fragment (e.g., terpenes and other compounds)	5.61	5.64	4.53
43.050	C_3_H_7_^+^	alkyl fragment (e.g., propene)	6.11	9.40	13.65
45.033	C_2_H_5_O^+^	acetaldehyde	3.24	3.94	1.55
47.012	CH_3_O_2_^+^	formic acid/formates	1.66	2.06	0.61
47.049	C_2_H_7_O^+^	ethanol	0.19	0.00	0.07
51.044	CH_7_O_2_^+^	n.a.	0.00	0.00	0.01
53.002	C_2_H_2_N^+^	n.a.	0.08	0.11	0.06
53.038	C_4_H_5_^+^	C4 fragment	0.13	0.17	0.14
55.054	C_4_H_7_^+^	C4 aldehydes fragment	2.20	2.49	2.49
57.033	C_3_H_5_O^+^	alkyl fragment (e.g., hexanal/1-butanol/1-octanol)	1.83	1.66	1.12
57.069	C_4_H_9_^+^	alkyl fragment (hexanol/valeric acid)	11.94	13.33	7.46
59.049	C_3_H_7_O^+^	acetone	9.66	6.41	8.60
61.028	C_2_H_5_O_2_^+^	acetic acid	21.66	3.16	2.08
63.001	CH_3_O_3_^+^	n.a.	0.00	0.00	0.04
67.054	C_5_H_7_^+^	terpenes fragments	0.12	0.16	0.16
69.069	C_5_H_9_^+^	isoprene/terpenes fragments	0.38	0.55	0.46
71.049	C_4_H_7_O^+^	2-butenal	0.23	0.51	0.72
71.086	C_5_H_11_^+^	3-methyl-1-butanol/alcohol fragment	0.23	0.28	0.22
73.028	C_3_H_5_O_2_^+^	n.a.	0.21	0.27	0.05
73.064	C_4_H_9_O^+^	2-butanone/isobutyraldehyde	0.19	0.26	0.19
75.044	C_3_H_7_O_2_^+^	methyl acetate/propanoates	0.17	0.34	0.14
77.059	C_3_H_9_O_2_^+^	propylene glycol	0.08	0.16	0.07
79.054	C_6_H_7_^+^	C6 fragments	0.05	0.08	0.09
81.069	C_6_H_9_^+^	C6 and terpenes fragments	0.77	1.05	1.63
83.049	C_5_H_7_O^+^	3-methyl furan	0.11	0.21	0.10
83.086	C_6_H_11_^+^	C6 compounds/hexenol fragment	0.26	0.28	0.43
85.065	C_5_H_9_O^+^	methyl-butenal/pentenone	0.39	0.60	0.22
85.101	C_6_H_13_^+^	alcohol (1-hexanol/nonanol)	0.18	0.40	0.30
87.044	C_4_H_7_O_2_^+^	2,3-butandione/diacetyl	0.32	0.44	0.07
87.080	C_5_H_11_O^+^	pentanal/methylbutanal	0.61	0.70	0.12
89.059	C_4_H_9_O_2_^+^	ethyl acetate/methylpropanoate/alkyl fragment	0.06	0.09	0.05
91.054	C_7_H_7_^+^	monoterpene fragments (e.g., thujone, linalool)	0.06	0.07	0.07
93.069	C_7_H_9_^+^	terpenes fragments	0.40	0.38	0.37
95.049	C_6_H_7_O^+^	phenol	0.09	0.44	0.10
95.086	C_7_H_11_^+^	terpenes fragments	0.14	0.36	0.17
97.069	C_5_H_5_O_2_^+^	dimethyl-furan	0.08	0.08	0.04
99.080	C_6_H_11_O^+^	hexenals/methylpentenone	0.07	0.12	0.10
101.060	C_5_H_9_O_2_^+^	hexanal	0.11	0.14	0.05
101.096	C_6_H_13_O_2_^+^	pentanedione	0.20	0.21	0.00
103.075	C_5_H_11_O_2_^+^	butyl formate	0.02	0.06	0.03
105.075	C_8_H_9_^+^	styrene	0.00	0.03	0.02
107.049	C_7_H_7_O^+^	benzaldehyde	0.07	0.06	0.05
107.086	C_8_H_11_^+^	terpenes fragment	0.04	0.09	0.06
109.101	C_8_H_13_^+^	sesqui/terpenes fragments	0.10	0.19	0.18
111.080	C_7_H_11_O^+^	dimethyl-2-cyclopenten-1-one	0.04	0.08	0.02
113.095	C_7_H_13_O^+^	n.a.	0.04	0.07	0.02
115.075	C_6_H_11_O_2_^+^	*γ*-hexalactone	0.04	0.05	0.02
117.091	C_6_H_13_O_2_^+^	hexanoic acid/methyl valerate	0.00	0.02	0.01
119.085	C_9_H_11_^+^	terpenes fragments	0.02	0.04	0.02
121.101	C_9_H_13_^+^	sesquiterpene fragments	0.02	0.16	0.03
123.116	C_9_H_15_^+^	sesquiterpene fragments	0.02	0.05	0.03
125.101	C_8_H_13_O^+^	(E)-2-octenal	0.02	0.05	0.03
127.111	C_8_H_15_O^+^	1-octen-3-one	0.02	0.05	0.03
129.127	C_8_H_17_O^+^	methyl hexyl ketone	0.02	0.04	0.02
131.086	C_10_H_11_^+^	terpene oxidation products	0.00	0.00	0.02
133.111	C_10_H_13_^+^	*p*-cymenene/fragments	0.00	0.03	0.02
135.125	C_10_H_15_^+^	*p*-cymene	0.02	0.18	0.08
136.033	n.a.	n.a.	0.00	0.32	0.04
137.132	C_10_H_17_^+^	monoterpenes	0.28	0.60	0.60
139.113	C_9_H_14_O^+^	nopinone/isophorone	0.01	0.02	0.04
141.132	C_10_H_21_^+^	terpenes fragments	0.00	0.02	tr
143.118	C_8_H_15_O_2_^+^	lactone compound	0.09	0.11	0.10
145.123	C_8_H_17_O_2_^+^	hexyl acetate/ethyl hexanoate	0.00	0.00	0.01
147.080	C_10_H_11_O^+^	n.a.	0.00	0.00	0.01
149.132	C_11_H_17_^+^	sesquiterpene fragments	0.00	0.03	0.07
151.111	C_10_H_15_O^+^	monoterpenes oxygenate (e.g., carvone)	0.00	0.08	0.02
153.127	C_10_H_17_O^+^	terpenoid-like compound (e.g., camphor, fenchone)	0.11	0.26	0.13
155.143	C_10_H_19_O^+^	alcohol monoterpenes (e.g., sabinene hydrate)	0.00	0.00	0.01
157.158	C_10_H_21_O^+^	terpenoids (e.g., menthol)	0.00	0.01	tr
159.140	C_9_H_19_O_2_^+^	C9 ester/octyl formate	0.00	0.00	tr
161.132	C_12_H_17_^+^	terpene oxidation products	0.00	0.00	0.01
163.145	C_12_H_19_^+^	sesquiterpene fragments	0.00	0.00	0.02
165.145	C_12_H_21_^+^	terpene oxidation products	0.00	0.02	0.01
177.184	C_10_H_25_O^+^	n.a.	0.00	0.01	tr
189.185	C_11_H_25_O_2_^+^	1,11-undecanediol	0.00	0.00	tr
203.79	C_15_H_23_^+^	sesquiterpenes (e.g., curcumene, calamene) and terpene fragments	0.00	0.00	tr
205.195	C_15_H_25_^+^	sesquiterpenes	0.02	0.03	0.05
217.160	C_15_H_21_O^+^	aromatic terpenoid (e.g., curzerene/furanodiene)	0.00	0.00	0.01
221.189	C_15_H_25_O^+^	sesquiterpenes alcohol (e.g., caryophyllene oxide)	0.00	0.01	tr
231.230	C_14_H_31_O_2_^+^	n.a.	0.00	0.01	0.00
		number of signals detected	72	61	83
		total VOCs emission (ppbv)	6184.26	5643.04	16,296.73
		terpenes total emission (ppbv)	270.34	142.15	688.84

*m*/*z*: mass/charge ratios; chemical formula for each VOCs species: ^1^ tentative identification of each signal detected. The terpene identification was based on pattern fragmentations proposed for the PTR instrument. 0.00: not detected; n.a.: identification not assigned; tr: very low signal strength; detection limit = 0.5 ppb.

## Data Availability

All generated data are included in this article.

## Supplementary Materials

The supporting information can be downloaded at: https://www.mdpi.com/article/10.3390/molecules27248739/s1. Figure S1: Spectra image.

## Sample Availability

Samples of the compounds of pollen *C. sativa* L. are available from the authors.
