# Supplementary figures and images for: SPME-GC-MS and PTR-ToF-MS Techniques for the Profiling of the Metabolomic Pattern of VOCs and GC-MS for the Determination of the Cannabinoid Content of Three Cultivars of Cannabis sativa L. Pollen
Source: Molecules. 2022 Dec 9;27(24):8739. doi: 10.3390/molecules27248739 (PMC9784944; doi:10.3390/molecules27248739)

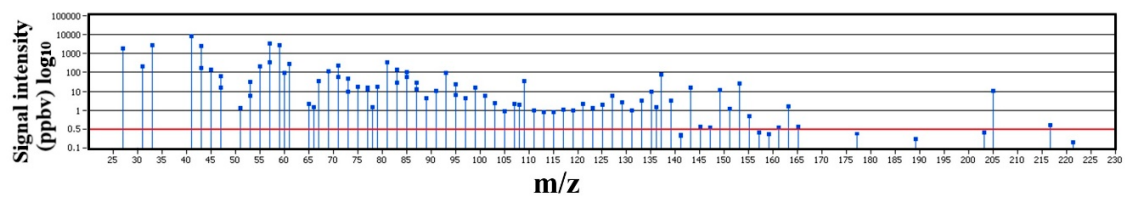

Figure S1: Spectra image.

Supplement: Supplementary file 1 [file molecules-27-08739-s001.zip › molecules-2033074-supplementary.pdf]
